# 2469: Streamlining study design and statistical analysis for quality improvement and research reproducibility

**DOI:** 10.1017/cts.2017.78

**Published:** 2018-05-10

**Authors:** Ram Gouripeddi, Mollie Cummins, Randy Madsen, Bernie LaSalle, Andrew Middleton Redd, Angela Paige Presson, Xiangyang Ye, Julio C. Facelli, Tom Green, Steve Harper

**Affiliations:** The University of Utah School of Medicine, Salt Lake City, UT, USA

## Abstract

OBJECTIVES/SPECIFIC AIMS: Key factors causing irreproducibility of research include those related to inappropriate study design methodologies and statistical analysis. In modern statistical practice irreproducibility could arise due to statistical (false discoveries, p-hacking, overuse/misuse of *p*-values, low power, poor experimental design) and computational (data, code and software management) issues. These require understanding the processes and workflows practiced by an organization, and the development and use of metrics to quantify reproducibility. METHODS/STUDY POPULATION: Within the Foundation of Discovery – Population Health Research, Center for Clinical and Translational Science, University of Utah, we are undertaking a project to streamline the study design and statistical analysis workflows and processes. As a first step we met with key stakeholders to understand the current practices by eliciting example statistical projects, and then developed process information models for different types of statistical needs using Lucidchart. We then reviewed these with the Foundation’s leadership and the Standards Committee to come up with ideal workflows and model, and defined key measurement points (such as those around study design, analysis plan, final report, requirements for quality checks, and double coding) for assessing reproducibility. As next steps we are using our finding to embed analytical and infrastructural approaches within the statisticians’ workflows. This will include data and code dissemination platforms such as Box, Bitbucket, and GitHub, documentation platforms such as Confluence, and workflow tracking platforms such as Jira. These tools will simplify and automate the capture of communications as a statistician work through a project. Data-intensive process will use process-workflow management platforms such as Activiti, Pegasus, and Taverna. RESULTS/ANTICIPATED RESULTS: These strategies for sharing and publishing study protocols, data, code, and results across the spectrum, active collaboration with the research team, automation of key steps, along with decision support. DISCUSSION/SIGNIFICANCE OF IMPACT: This analysis of statistical methods and process and computational methods to automate them ensure quality of statistical methods and reproducibility of research.

